# Identification and Characterization of Two Novel Compounds: Heterozygous Variants of *Lipoprotein Lipase* in Two Pedigrees With Type I Hyperlipoproteinemia

**DOI:** 10.3389/fendo.2022.874608

**Published:** 2022-07-18

**Authors:** Shuping Wang, Yiping Cheng, Yingzhou Shi, Wanyi Zhao, Ling Gao, Li Fang, Xiaolong Jin, Xiaoyan Han, Qiuying Sun, Guimei Li, Jiajun Zhao, Chao Xu

**Affiliations:** ^1^ Department of Endocrinology and Metabolism, Shandong Provincial Hospital, Cheeloo College of Medicine, Shandong University, Jinan, China; ^2^ Department of Endocrinology and Metabolism, Shandong Provincial Hospital Affiliated to Shandong First Medical University, Jinan, China; ^3^ Department of Endocrinology and Metabolism, Dongying People’s Hospital, Dongying, China; ^4^ Central Laboratory, Shandong Provincial Hospital Affiliated to Shandong First Medical University, Jinan, China; ^5^ Shandong Clinical Research Center of Diabetes and Metabolic Diseases, Jinan, China; ^6^ Shandong Key Laboratory of Endocrinology and Lipid Metabolism, Jinan, China; ^7^ Shandong Prevention and Control Engineering Laboratory of Endocrine and Metabolic Diseases, Jinan, China; ^8^ Department of Pediatrics, Shandong Provincial Hospital Affiliated to Shandong University, Jinan, China

**Keywords:** lipoprotein lipase (LPL), type 1, hypertriglyceridemia, variants, pedigree

## Abstract

**Background:**

Type I hyperlipoproteinemia, characterized by severe hypertriglyceridemia, is caused mainly by loss-of-function mutation of the *lipoprotein lipase* (*LPL*) gene. To date, more than 200 mutations in the *LPL* gene have been reported, while only a limited number of mutations have been evaluated for pathogenesis.

**Objective:**

This study aims to explore the molecular mechanisms underlying lipoprotein lipase deficiency in two pedigrees with type 1 hyperlipoproteinemia.

**Methods:**

We conducted a systematic clinical and genetic analysis of two pedigrees with type 1 hyperlipoproteinemia. Postheparin plasma of all the members was used for the LPL activity analysis. *In vitro* studies were performed in HEK-293T cells that were transiently transfected with wild-type or variant *LPL* plasmids. Furthermore, the production and activity of LPL were analyzed in cell lysates or culture medium.

**Results:**

Proband 1 developed acute pancreatitis in youth, and her serum triglycerides (TGs) continued to be at an ultrahigh level, despite the application of various lipid-lowering drugs. Proband 2 was diagnosed with type 1 hyperlipoproteinemia at 9 months of age, and his serum TG levels were mildly elevated with treatment. Two novel compound heterozygous variants of *LPL* (c.3G>C, p. M1? and c.835_836delCT, p. L279Vfs*3, c.188C>T, p. Ser63Phe and c.662T>C, p. Ile221Thr) were identified in the two probands. The postheparin LPL activity of probands 1 and 2 showed decreases of 72.22 ± 9.46% (p<0.01) and 54.60 ± 9.03% (p<0.01), respectively, compared with the control. *In vitro* studies showed a substantial reduction in the expression or enzyme activity of LPL in the *LPL* variants.

**Conclusions:**

Two novel compound heterozygous variants of *LPL* induced defects in the expression and function of LPL and caused type I hyperlipoproteinemia. The functional characterization of these variants was in keeping with the postulated *LPL* mutant activity.

## 1 Introduction

Type I hyperlipoproteinemia, also known as familial lipoprotein lipase (LPL) deficiency, is characterized by very severe hypertriglyceridemia with episodes of abdominal pain, recurrent acute pancreatitis, eruptive cutaneous xanthomata, and hepatosplenomegaly (1). The prevalence of type I hyperlipoproteinemia in the general population is estimated to be 1–2 per million (2, 3). Inherited in an autosomal recessive manner, type I hyperlipoproteinemia is caused mainly by the loss-of-function mutation of the *LPL* gene (4).

The *LPL* gene encodes a secreted glycoprotein, LPL, containing 448 amino acids (5). Synthesized by cells such as fat cells, macrophages, and muscle cells, LPL is an important rate-limiting enzyme for triglyceride degradation, which can hydrolyze triglycerides into fatty acids and glycerol to regulate lipid metabolism (6, 7). Defective *LPL* can cause the accumulation of triglyceride-rich chylomicrons and very low-density lipoproteins and further lead to severe hypertriglyceridemia. To date, more than 200 mutations in the *LPL* gene have been reported to result in type I hyperlipoproteinemia in the Human Gene Mutation Database (HGMD), while only a limited number of mutations have been evaluated for pathogenesis (8, 9).

In this study, two patients with severe hypertriglyceridemia were confirmed to have type I hyperlipoproteinemia by whole-exome sequencing (WES), and two novel compound heterozygous variants of the *LPL* gene were identified. We showed through bioinformatics analysis and *in vitro* experiments that these variants can affect the enzymatic activity, production, and/or secretion of LPL and cause type I hyperlipoproteinemia in both cases. Our research provides evidence for elucidating the molecular mechanisms of mutant *LPL* and helps improve the genetic diagnosis rate and precise treatment of this disease.

## 2 Materials and Methods

### 2.1 Ethics

This study was approved by the Ethics Committee of Shandong Provincial Hospital affiliated with Shandong University. The study protocol was in line with the Declaration of Helsinki (as revised in Brazil 2013). The consent obtained from all the participants was both informed and written.

### 2.2 Subjects and Follow-Up Studies

Two patients diagnosed with severe hypertriglyceridemia from our hospital were involved in the study. All patient data were collected at the first visit. All family members received specific physical and laboratory examinations in Shandong Provincial Hospital. Peripheral blood specimens were collected from each member for genetic analysis.

We followed these two patients from their first presence and closely tracked their clinical and biochemical information.

### 2.3 Whole-Exome Sequencing and Sanger Sequencing

Using a QIAamp DNA Mini Kit (Qiagen, Hilden, Germany), genomic DNA was isolated from peripheral blood leukocytes. Next, DNA from peripheral blood was used for WES. We performed genomic DNA fragmentation, paired-end adaptor ligation, amplification, and purification and then captured human exons using a SeqCap EZ Med Exome Enrichment Kit (Roche NimbleGen, Madison, WI, USA). By postcapture amplification and purification, a DNA library was generated and then sequenced with the Illumina HiSeq sequencing platform. To obtain the coverage and mean read depth of target regions, sequence data alignment to the human genome reference (hg19) and variant calling were performed using NextGene V2.3.4 software. The average coverage of the exome was >100×, which allowed a deep examination of the target region to accurately match >99% of the target exons. Mutations with low coverage in the target area were screened and filtered to ensure the accuracy of data analysis.

In addition, the frequency of normal populations [data from Genome Aggregation Database (GnomAD), Exome Aggregation Consortium (ExAC), Trans-Omics for Precision Medicine (TOPMED), Human Gene Mutation Database (HGMD), Clinvar and Online Mendelian Inheritance in Man (OMIM) databases] was obtained by NextGene V2.3.4 and our in-house scripts. A variant was identified as a mutation when it was not found in 500 Chinese controls, in dbSNP (http://www.ncbi.nlm.nih.gov/snp/) and the exome variant server (http://evs.gs.washington.edu/EVS/), or when the allele frequency was <0.001 in the database. According to Standards and Guidelines for the Interpretation of Sequence Variants published by the American College of Medical Genetics (ACMG) in 2015, pathogenic variants were determined with the Human Genome Variation Society (HGVS) nomenclature.

Candidate variants were detected by WES. Pathogenic or suspected pathogenic variants were verified by Sanger sequencing. Universally tagged sequencing primers were designed by using Primer3 version 1.1.4 (http://www.sourceforge.net) and GeneDistiller 2014 (http://www.genedistiller.org/). Polymerase chain reaction (PCR) was performed in a 50-μl system including 5 μl 10 × PCR buffer, 4 μl dNTPs, 4 μl genomic DNA, 1 μl forward and reverse primers, and 0.3 μl Taq Hot Start (Takara Bio, Otsu, Japan). The PCR conditions were as previously described (10). Amplicons were sequenced using an ABI 3730 system (Applied Biosystems, Foster City, CA, USA) and were analyzed by the autoassembler software Chromas 2.6 and visual inspection.

### 2.4 The Activity of LPL in Plasma

Preheparin blood samples were taken after fasting overnight. Then, heparin was injected intravenously (60 IU/kg), and the postheparin blood was collected from the contralateral arm 10 min later. The subjects stopped taking insulin and other medications in the morning to eliminate the interference caused by medications. The blood sample was centrifuged at 3,000 rpm for 10 min at 4°C to obtain plasma. A Lipoprotein Lipase Activity Assay Kit (Fluorometric, Biovision, Milpitas, CA, USA) was used to measure LPL activity. Mouse (C57BL/6) postheparin plasma was used as a positive control.

### 2.5 *In Vitro* Functional Analysis of *LPL* Variants

#### 2.5.1 Construction of Overexpression Plasmids Containing the Target *LPL* Mutation

By GeneArt Gene Synthesis (Thermo Fisher Scientific, Rockford, IL, USA), wild-type *LPL* cDNA (pcDNA3.1- *LPL*-WT) was synthesized and cloned into the pcDNA3.1 vector with a V5 epitope tag. *LPL* variants (pcDNA3.1-*LPL*-MU: p. M1?, p. L279Vfs*3, p. Ser63Phe, or p. Ile221Thr) were obtained by site-directed mutagenesis. To obtain the mutant *LPL* cDNA, we used the following primers: forward (wild type) GACCCAATAAGCTTCGTCAGAATTTTGTAATACGACTCACTATAGG, reverse (wild type) ATTGGGTCAAGCTTATGTTTTGTAAAAGTTACTTCCTCCACT; forward (p. M1)? CAGAGGGACGCGCCCCGAGATCGAGAGCAAAGCCCTGCTCGTGC, primer reverse (p. M1)? AGCACGAGCAGGGCTTTGCTCTCGATCTCGGGGCGCGTCCCTCTG; primer forward (p. L279Vfs*3) GACCCAATAAGCTTCGTCAGAATTTTGTAATACGACTCACTATAGG, primer reverse (p. L279Vfs*3) ATTGGGTCAAGCTTGGAACTGCACCTGTAGGCCTTACTTGGA; primer forward (p. Ser63Phe) CACCTCATTCCCGGAGTAGCAGAGTTCGTGGCTACCTGTCATTTCA, primer reverse (p. Ser63Phe) TGAAATGACAGGTAGCCACGAACTCTGCTACTCCGGGAATGAGGTG; primer forward (p. Ile221Thr) GAGGGTCCCCTGGTCGAAGCACTGGAATCCAGAAACCAGTTGG, primer reverse (p. Ile221Thr) CCCAACTGGTTTCTGGATTCCAGTGCTTCGACCAGGGGACCCTCT. All the primers used were purchased from GenScript (Cayman Islands, UK). Polymerase chain reaction (PCR) was carried out in a 50-μl reaction system including 5 μl 10 × PCR buffer, 4 μl genomic DNA, 4 μl dNTPs, 1 μl forward and reverse primers, and 0.3 μl Taq Hot Start (Takara Bio, Otsu, Japan). The PCR conditions consisted of an initial denaturation step (95°C for 5 min), followed by 40 cycles of denaturation (95°C, 30 s), annealing (55°C, 30 s), and elongation (68°C, 30 s). The presence and fidelity of pcDNA3.1-*LPL*-MU were confirmed by the ABI 3730 sequencing system (Applied Biosystems, Foster City, CA, USA).

#### 2.5.2 Transient Transfection

In high-glucose Dulbecco’s modified Eagle’s medium (DMEM) containing 10% fetal bovine serum (FBS), 5% penicillin–streptomycin, and 2 mM L-glutamine, human embryonic kidney 293T/17 (HEK293T/17) cells obtained from the National Collection of Authenticated Cell Cultures were cultured. Next, HEK293T/17 cells were transiently transfected with plasmids containing pcDNA3.1- *LPL*-WT and pcDNA3.1- *LPL*-MU by using Lipofectamine 3000 transfection reagent (Thermo Fisher Scientific). As previously described (10), the transfection concentration of a single plasmid was 2.5 μg/ml, and the concentration of each plasmid was 1.5 μg/ml at the cotransfection of the two plasmids.

After 48 h, the cells were lysed with mammalian protein extraction reagent (Thermo Fisher Scientific) containing protease inhibitor cocktail (Sigma-Aldrich). HEK293T/17-cell lysates were used to analyze the protein expression of LPL by Western blotting. We used Amicon^®^ Ultra15 ultrafiltration centrifuge tubes (Millipore-Sigma, Burlington, MA, USA) to concentrate the medium 80 times. The concentrated medium was used for LPL activity analysis.

#### 2.5.3 The Activity of LPL in Cell Culture Medium

The Lipoprotein Lipase Activity Assay Kit (Fluorometric, Biovision) was used to determine LPL activity in the culture medium of HEK293T/17 cells transfected with *LPL* wild-type plasmids and plasmids containing *LPL* variants.

#### 2.5.4 Immunoblotting

We boiled the cell lysates mixed with Laemmli buffer containing 2-mercaptoethanol for 5 min at 99°CC. Proteins were separated by sodium dodecyl sulfate–polyacrylamide gel electrophoresis (SDS-PAGE; 100 V, 90 min) and then transferred onto a nitrocellulose membrane (400 mA, 1 h). Membranes were incubated overnight with primary antibodies and washed three times for 10 min with 0.2% Tris-buffered saline Tween (TBST). Then, the membranes were incubated with secondary antibodies for 1 h and washed three times for 10 min with 0.2% TBST. Next, a 5-min incubation between chemiluminescent horseradish peroxidase (HRP) substrate (Millipore Corporation, Billerica, MA, USA) and membranes was conducted, and bands were visualized by a ChemiDoc XRS System (Bio-Rad, Hercules, CA, USA). The following antibodies were used: rabbit monoclonal anti-LPL antibody (Abcam Ab91606) and mouse monoclonal anti-GAPDH (glyceraldehyde-3-phosphatee dehydrogenase) antibody (Abcam Ab8245).

### 2.6 Statistical Analysis

Statistical analysis was performed using the SPSS 24.0 software package (SPSS Inc., Chicago, IL, USA). The Kolmogorov–Smirnov test was used to determine the distribution of continuous variables. Continuous variables with a normal distribution are given as the mean ± standard deviation (SD) and were compared by the Mann–Whitney U test. The results were considered statistically significant when the p-value was <0.05.

## 3 Results

### 3.1 Clinical Manifestations

**Figure 1:**
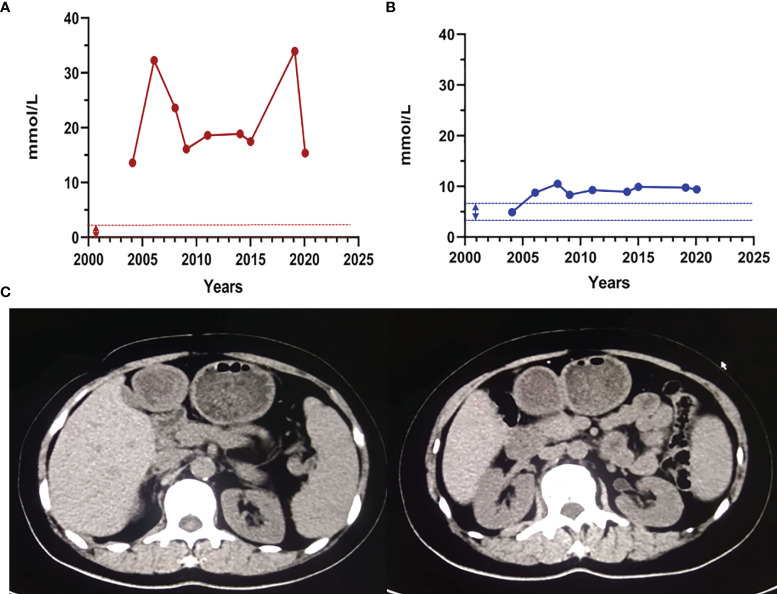
The clinical characteristics of proband 1. **(A)** The proband 1 triglyceride and total cholesterol levels. The dashed line indicates the normal reference range. Abbreviations: TG, triglycerides; TC, total cholesterol. Normal range: TG: 0.4–1.8 mmol/L; TC: 3.6–6.2 mmol/L. **(B)** Abdomen and lower limbs of patient 1. **(C)** Abdominal CT scan of patient 1. The pancreas was irregular in shape; the liver was normal in shape and size; no abnormal density shadow was observed in the parenchyma; no expansion of the bile ducts in and outside the liver was observed; the shape and size of the gallbladder were fine; no abnormal density shadows were found in the gallbladder; and the spleen was normal in shape and size.

**Figure 2:**
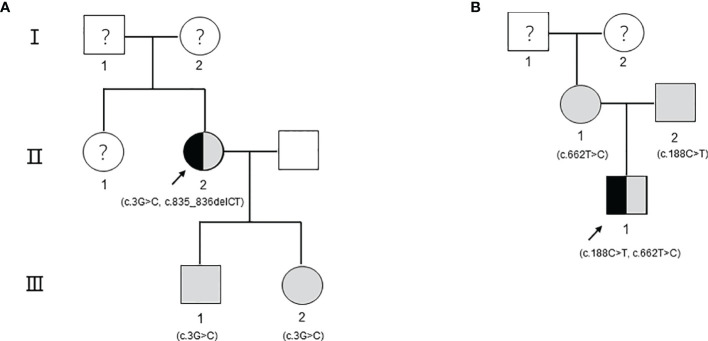
The pedigree including the patients. The arrow refers to the proband. Black indicates that the person has type I hyperlipoproteinemia. The shading indicates that the person carries gene mutations but has a healthy phenotype. Circles indicate female individuals reared, and squares indicate male individuals reared. The question mark means that it cannot be determined whether the individual carries a genetic mutation. **(A)** The pedigree of patient 1. **(B)** The pedigree of patient 2.

Proband 1, female, was hospitalized with acute necrotizing pancreatitis and underwent abdominal surgery at the age of 24. At 25 years old, she was found to have high triglyceride (TG) levels (approximately 17 mmol/L). However, TG levels increased further after she was administered some lipid-lowering drugs (including fenofibrate, rosuvastatin, Chinese medicine, etc.). In recent years, TG levels fluctuated at 13.63–33.94 mmol/L, and TC levels fluctuated at 4.92–10.51 mmol/L after drug withdrawal (**Figures 1A, B**). At the age of 49, the proband presented symptoms of hyperglycemia with fasting plasma glucose at 15.22 mmol/L and developed vomiting and diarrhea after oral administration of Chinese medicine decoction. Abdominal computerized tomography (CT) scan (**Figure 1C**) showed that the pancreas was irregular in shape; no obvious abnormality was observed in other parts. After giving patient 1 the low-fat diet, insulin, and liver protection drugs, the last follow-up in June 2020 showed that the TG, TC, and fasting plasma glucose were 15.37, 9.43, and 6.01 mmol/L, respectively (**Table 1**). The family history of the patient showed that the patient’s parents and her sister were deceased and could not be tested. However, it was recalled that her sister had also suffered from recurrent pancreatitis (**Figure 2A**).

**Table 1:** Clinical features and lipoprotein profile of the individuals screened for *LPL* variants.

Variable	Patient 1	Patient 2
Age (years)	49	5
Gender (male/female)	Female	Male
BMI (kg/m^2^)	18.8	17.6
Alcohol intake (yes/no)	No	No
Diabetes mellitus (yes/no)	Yes	No
History of pancreatitis (yes/no)	Yes	No
Number of pancreatitis	2	0
Pregnancy	Yes	No
TG (mmol/L)	15.37	3.35
TC (mmol/L)	9.43	2.72
HDL-c (mmol/L)	0.57	0.75
LDL-c (mmol/L)	3.30	1.42
FBG (mmol/L)	6.01	5.60
Treatment	Low-fat diet, statin, fibrate, Chinese medicine, insulin, and liver protection drugs	Low-fat diet, insulin

BMI, body mass index; TG, triglycerides; TC, total cholesterol; HDL-c, high-density lipoprotein cholesterol; LDL-c, low-density lipoprotein cholesterol; FBG, fasting blood glucose. Normal range: TG: 0.4–1.8 mmol/L; TC: 3.6–6.2 mmol/L; HDL-c: 0.8–1.5 mmol/L; LDL-c: 0.5-3.36; FBG, 3.9–6.3 mmol/L.

Proband 2 came to our hospital for treatment because his blood sample showed severe hyperlipemia during the physical examination at 9 months old. On the second day of admission, his TG level was 20.35 mmol/L, and his TC level was 8.89 mmol/L. A low-fat diet and 4 U of continuous intravenous infusion of insulin were ordered to lower blood lipids. After 3 days, the TG level was 22.77 mmol/L, and the TC was 7.60 mmol/L. After 7 days, the TG level was 12.32 mmol/L, and the TC level was 4.55 mmol/L. After 10 days, the proband was discharged, his blood lipids improved significantly, with TG at 10.25 mmol/L and TC at 4.93 mmol/L. After being discharged from the hospital, he continued to consume a low-fat diet without any special discomfort. The last follow-up conducted in June 2020 showed that the serum TG was 3.35 mmol/L, the TC was 2.72 mmol/L, and the fasting plasma glucose was 5.60 mmol/L (**Table 1**). The family history of the patient shows that the parents of patient 2 have thus far been healthy (**Figure 2B**).

### 3.2 Variant Detection

**Figure 3:**
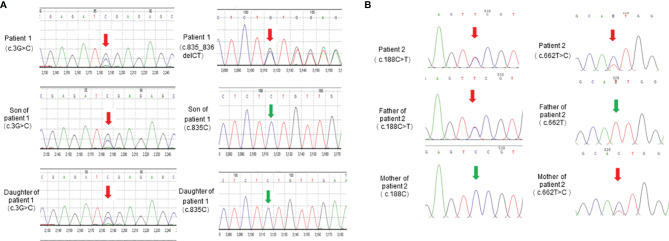
*LPL* gene sequencing diagram. **(A)** *LPL* gene (reference sequence NM_000237) sequencing diagram of patient 1 and her children. **(B)** *LPL* gene (reference sequence NM_000237) sequencing diagram of patient 2 and his parents.

To further identify disease-causing genes to facilitate diagnosis, we subsequently applied the WES technique for genetic analysis of the two pedigrees. According to HGMD, we found a novel compound variant of *LPL* (c.3G>C and c.835_836delCT, **Figure 3A**) in proband 1, which may cause truncation mutations (p. M1)?, and frameshift mutations (p. L279Vfs*3) leading to premature amino acid stop codes for protein synthesis, respectively. The p. M1? variant has been reported in a related clinical case, and its region is the translation initiation codon of this protein (11). The p. L279Vfs*3 variant is absent in the GnomAD, TOPMED, and ExAC databases. Notably, the son and daughter of proband 1 carry the p. M1? variant derived from the maternal line, as verified by Sanger sequencing, but their lipid levels and other phenotypes were normal.

We also identified proband 2 as carrying a novel compound variant of *LPL* (c.188C>T and c.662T>C, **Figure 3B**) that resulted in a change in the 63rd amino acid of the encoded protein from serine to phenylalanine (p. Ser63Phe) and a change in the 221st amino acid of the encoded protein from isoleucine to threonine (p. Ile221Thr), respectively. The p. Ser63Phe variant is absent in the GnomAD, TOPMED, and ExAC databases, while the p. Ile221Thr variant has been reported in three unrelated probands causing lipoprotein lipase deficiency (12). In addition, the parents of proband 2 each carried a variant. The Ser63Phe variant comes from the father, and the p. Ile221Thr variant comes from the mother, but they have thus far been healthy.

### 3.3 The Activity of LPL in Plasma

**Figure 4:**
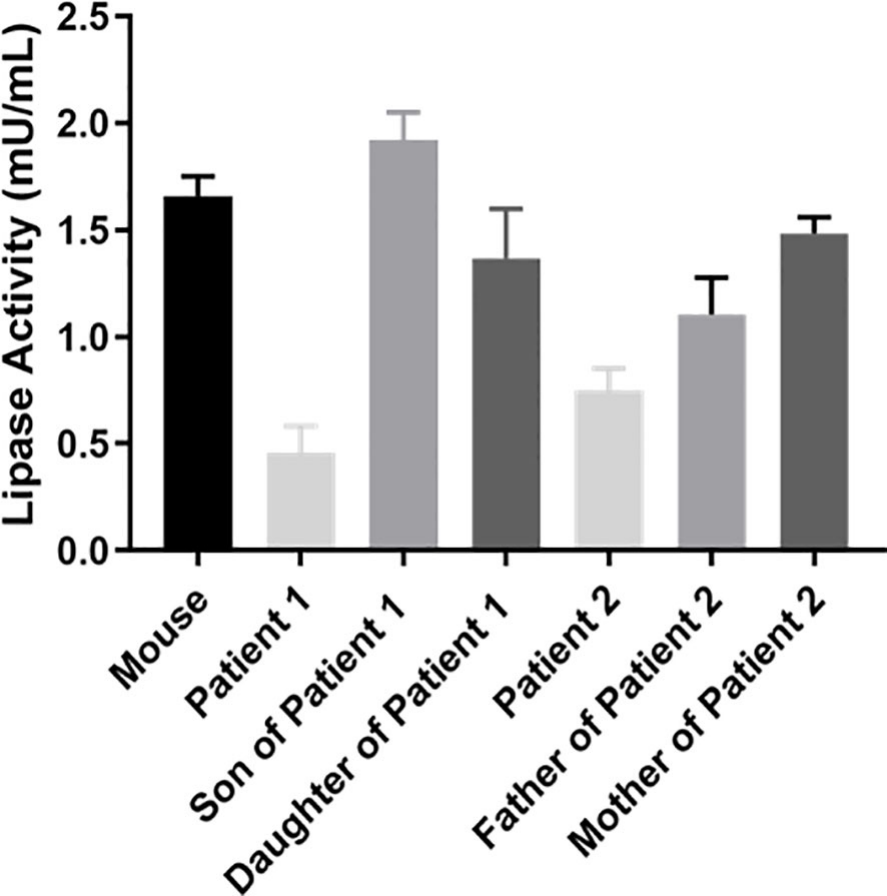
Analysis of LPL activity in plasma. Peripheral blood was collected at 10 min after heparin injection (60 IU/kg) to assay lipase activity by the enzyme-fluorescent method.

Accordingly, the postheparin LPL activity of patient 1 (0.46 ± 0.13 mU/ml) and patient 2 (0.75 ± 0.11 mU/ml) actually showed a decrease of 72.22 ± 9.46% (p<0.01) and 54.60 ± 9.03% (p<0.01), respectively, compared with the control (1.66 ± 0.09 mU/ml). The differences between the son and daughter of proband 1, the parents of proband 2, and the control seem to be negligible (**Figure 4**). Therefore, two novel compound heterozygous variants of *LPL* may cause changes in LPL enzyme activity in the patient’s plasma, leading to defects in triglyceride metabolism and resulting in extremely elevated serum triglycerides.

### 3.4 LPL Protein Expression in Cells

**Figure 5:**
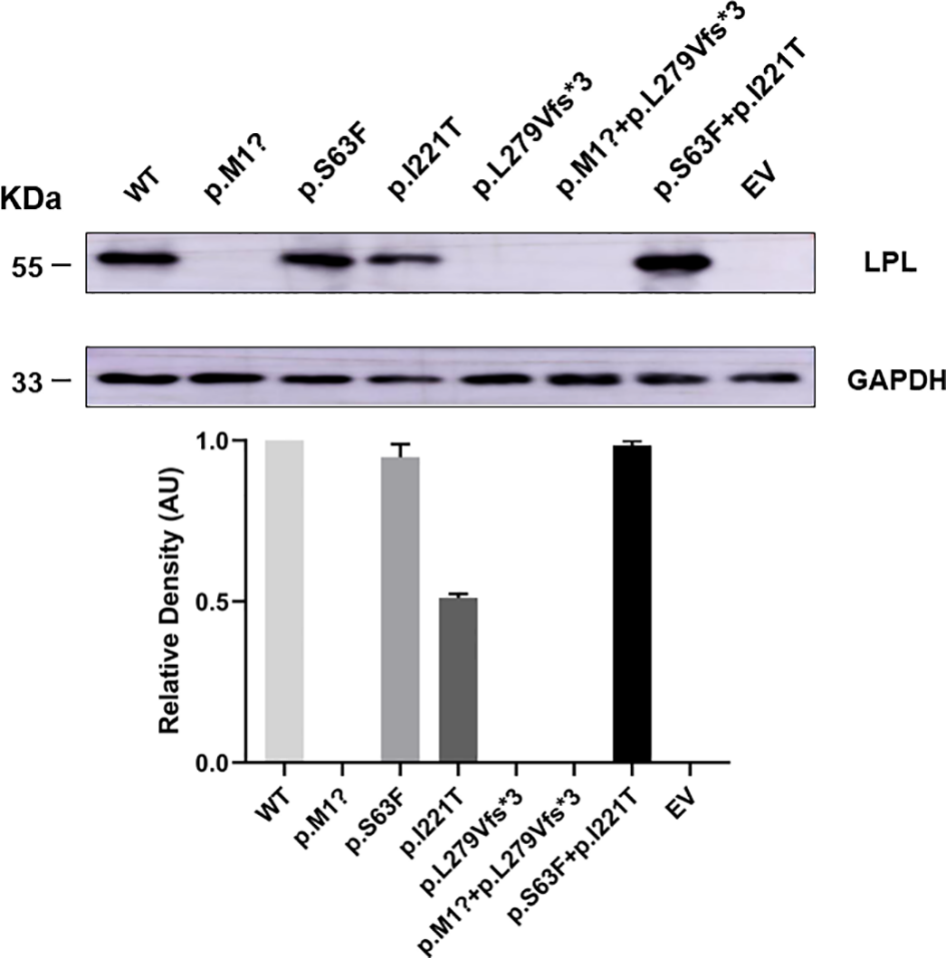
HEK 293T/17 cells transiently transfected showed a reduction in the production of *LPL* variants. HEK293T/17 cells transfected with the p. M1? mutation, p. L279Vfs*3 mutation, or p. M1? and p. L279Vfs*3 mutation showed virtually no protein in the cell lysate (p<0.01). Compared with cells transfected with the plasmids containing the pcDNA3.1-*LPL*-WT, there was no significant difference in the amount of protein reduction produced by the cells transfected with the p. S63F mutation or the two variants (p. S63F and p. I221T), but protein synthesis was reduced by 48.33 ± 1.78% in cells transfected with the p. I221T variant (p<0.01). Abbreviations: EV, empty vector; WT, wild type; LPL, lipoprotein lipase.

To understand the effect of the identified *LPL* variants at the protein level, we transiently transfected the plasmids containing pcDNA3.1-*LPL*-WT and pcDNA3.1-*LPL*-MU into HEK293T/17 cells. HEK293T/17 cells transfected with the p. M1? mutation, p. L279Vfs*3 mutation, or p. M1? and p. L279Vfs*3 mutation showed virtually no protein in the cell lysate (p<0.01, **Figure 5**). Compared with cells transfected with the plasmids containing the pcDNA3.1-*LPL*-WT, there was no significant difference in the amount of protein reduction produced by the cells transfected with the p. S63F mutation or the two variants (p. S63F and p. I221T), but protein synthesis was reduced by 48.33 ± 1.78% in cells transfected with the p. I221T variant (p<0.01, **Figure 5**).

### 3.5 The Activity of LPL in Cell Culture Medium

**Figure 6:**
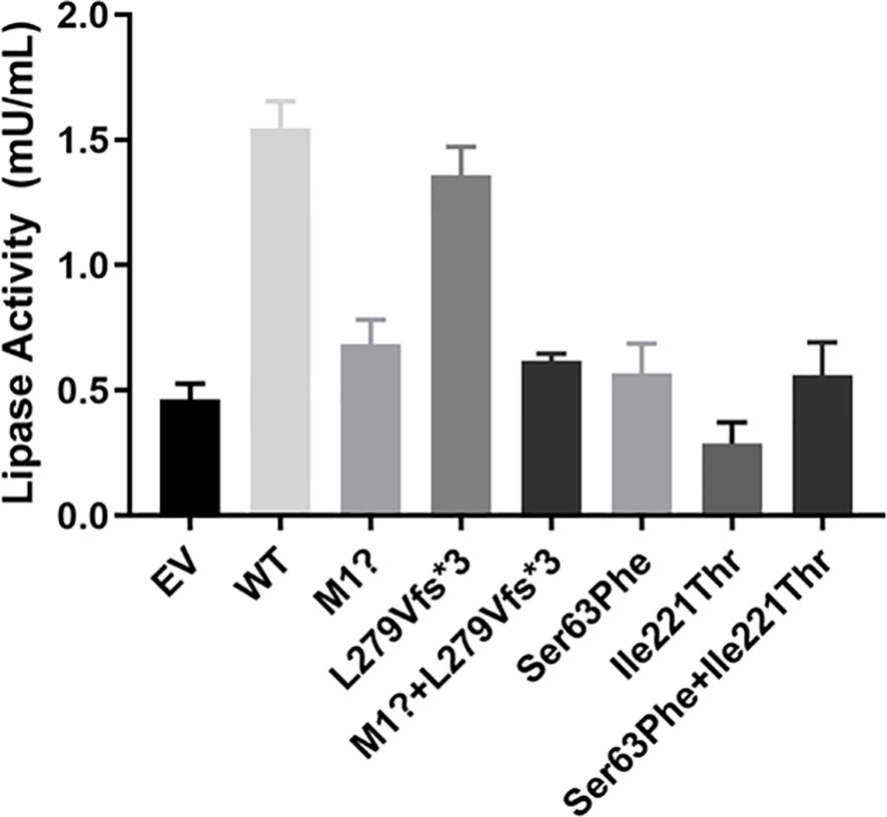
Functional analysis of *LPL* mutants in the medium. Except for the p. L279Vfs*3 mutation, LPL activity in the medium transfected with other plasmids containing pcDNA3.1-*LPL*-MU was significantly reduced compared with the wild type. Specifically, cells transfected with the p. M1? or the two variants (p. M1? and p. L279Vfs*3) showed reductions in protein activity of approximately 55.93 ± 3.28% (p<0.01) or 59.84 ± 4.47% (p<0.01), while LPL activity was reduced by 62.76 ± 9.90% (p<0.01) or 63.25 ± 10.55% (p<0.01) in the media of HEK 293T/17 cells transfected with p. Ser63Phe or the two variants (p. Ser63Phe and Ser63Phe+Ile221Thr). The media of cells transfected with p. Ile221Thr demonstrated a maximum reduction in LPL activity of approximately 81.56 ± 4.35% (p<0.01).

Next, we tested LPL activity in media of HEK 293T/17 cells. Wild-type LPL medium was used as a positive control. Except for the p. L279Vfs*3 mutation, LPL activity in the medium transfected with other plasmids containing pcDNA3.1-*LPL*-MU was significantly reduced compared with the LPL activity of the wild type. Specifically, cells transfected with the p. M1? or the two variants (p. M1? and p. L279Vfs*3) showed reductions in protein activity of approximately 55.93 ± 3.28% (p<0.01) or 59.84 ± 4.47% (p<0.01), while LPL activity was reduced by 62.76 ± 9.90% (p<0.01) or 63.25 ± 10.55% (p<0.01) in the media of HEK 293T/17 cells transfected with p. Ser63Phe or the two variants (p. Ser63Phe and Ser63Phe+Ile221Thr). The media of cells transfected with p. Ile221Thr demonstrated a maximum reduction in LPL activity of approximately 81.56 ± 4.35% (p<0.01, **Figure 6**).

## 4 Discussion

In the present study, we systematically identified and characterized two novel compound heterozygous variants of the *LPL* gene in patients with type I hyperlipoproteinemia. This harmful blood lipid metabolism disorder prompted us to solve this problem by determining the activity of LPL in plasma after heparin and analyzing the activity and quality defects of LPL *in vitro.* The functional characterization of these variants was in keeping with the postulated *LPL* mutant activity.

LPL is the rate-limiting enzyme in normal triglyceride metabolism, which plays a central role in the hydrolysis of triglycerides present in very-low-density lipoproteins and chylomicrons (13). Therefore, defective LPL can cause severe hypertriglyceridemia, such as type I hyperlipoproteinemia, a rare autosomal recessive genetic disease (14). In patients with hypertriglyceridemia, low plasma LPL activity and quality have been found (4, 15), and this phenomenon is usually caused by pathogenic *LPL* variants (16–18). The LPL protein consists of a large N-terminal domain (amino acid residues 1–315) and a small C-terminal domain (amino acid residues 316–448). The N-terminal domain contains the catalytic center, active site region, substrate-binding site, and heparin-binding site (19, 20). In addition to participating in the binding of heparin to the substrate, the C-terminal domain has been considered to be critical to the formation and stability of the LPL head and tail non-covalent homodimers (21, 22). Although LPL has always been considered to be active only as a homodimer, there are reports of biochemical data showing that LPL in complex with GPIHBP1 can be active as a monomer 1:1 complex (23).

Interestingly, we detected a novel compound variant of *LPL* (p. M1? and p. L279Vfs*3) in a Chinese patient with type I hyperlipoproteinemia. This patient exhibited severe hypertriglyceridemia and recurrent acute pancreatitis. In particular, the p. M1? variant, an initiation codon mutation, has previously been identified in an 18-year-old patient from China who also carried the previously reported heterozygous substitution of glutamic acid at residue 242 with lysine (p. Glu242Lys) (11). *In vitro* experiments found that the p. M1? had approximately 3% protein mass and 2% activity, whereas p. Glu242Lys had normal mass but undetectable activity; that is, the p. M1? variant rather than the heterozygous p. Glu242Lys variant is mainly responsible for the phenotypic expression of type I hyperlipoproteinemia in this patient. Here, our *in vitro* expression of both mutations separately or in combination displayed virtually no protein in the cell lysate. Except for the p. L279Vfs*3 mutation, the LPL activity in the medium transfected with p. M1? or both mutations in combination was significantly reduced compared with the LPL activity of the wild type. Therefore, we speculate that the p. M1? mutation will have a significant impact on the quality and activity of LPL, but p. L279Vfs*3 has little effect on the activity. What needs illustration is that the son and daughter also carry the p. M1? variant derived from proband 1, but their lipid levels and other phenotypes were normal. Compared with the control, the postheparin LPL activity in the plasma of the son and daughter was not different. Hence, what caused proband 1 to have severe hyperlipidemia? This requires more in-depth research to explore.

Proband 2 also carries a novel compound variant of *LPL* (p. Ser63Phe and p. Ile221Thr). The p. Ile221Thr variant has been reported in three unrelated probands causing lipoprotein lipase deficiency (12). The synthesis and secretion of a catalytically defective protein induced by the p. Ile221Thr mutation were confirmed through *in vitro* experiments in COS-1 cells. In accordance with the previous data, two new compound *LPL* variants of patient 2 in our study caused changes in the LPL enzyme activity in the plasma of the patient, whereas the differences in enzyme activity between the parents of proband 2 and the control seemed to be negligible. Curiously, the parents each carried a variant of proband 2. Sanger sequencing confirmed that the Ser63Phe variant comes from the father and the p. Ile221Thr variant comes from the mother, but the parents have thus far been healthy. Our *in vitro* experiments to understand the effect of the two *LPL* variants at the protein level indicated that the p. I221T variant can reduce approximately 48.33 ± 1.78% mass and approximately 81.56 ± 4.35% activity, and the LPL activity was reduced by 62.76 ± 9.90% or 63.25 ± 10.55% in the media of HEK 293T/17 cells transfected with p. Ser63Phe or the two variants (p. Ser63Phe and Ser63Phe+Ile221Thr).

Functional studies have potential benefits for the diagnosis and treatment of severe hypertriglyceridemia and can promote the mechanistic study of the occurrence of type I hyperlipoproteinemia. Ultimately, the findings may lead to the development of more effective drugs for the precise intervention for such patient rather than limited symptomatic treatment. In this study, differences in the activity of LPL would affect the corresponding clinical research, and interested researchers may summarize more cases and find some rules, such as mutations in one exon region having a greater impact on LPL activity, while mutations in another exon region have little impact on LPL activity.

This study has some limitations. For instance, in our study, we could not analyze whether the parents of patient 1 had hypertriglyceridemia because they had passed away. Second, we have not been able to conduct a more in-depth study on why the son and daughter of proband 1 and the parents of proband 2 carry a certain variant, but the phenotype is normal. Our patients did not have two harmful lifestyle factors, namely, severe obesity and severe tobacco abuse. Obesity is not only related to primary hypertriglyceridemia but also a risk factor for secondary hypertriglyceridemia (24). Tobacco can affect fat metabolism in the liver and increase TG levels due to defects in the lipolysis system (25). In short, it can basically rule out the possibility that our two probands had secondary hypertriglyceridemia.

## 5 Conclusions

To conclude, we have described two novel compound heterozygous variants of *LPL* in patients with type I hyperlipoproteinemia, showing that all these variants are pathogenic by disrupting LPL mass and the enzymatic activity of LPL. Although *LPL* variants are rare, WES is important to discover the monogenic and oligogenic genetic patterns in severe hyperlipoproteinemia, a challenge now predigested by the access to next-generation sequencing.

## Data Availability Statement

The datasets presented in this study can be found in online repositories. The names of the repository/repositories and accession number(s) can be found below: GenBank, 2561205.

## Ethics Statement

The studies involving human participants were reviewed and approved by Ethics Committee of Shandong Provincial Hospital affiliated to Shandong University. Written informed consent to participate in this study was provided by the participants’ legal guardian/next of kin.

## Author Contributions

SW and YC designed the experiments, researched the data, and wrote the manuscript. YS contributed to the statistical analysis. LG and WZ contributed to the experiments and technical supporting. LF, XJ, XH, and QS to the data Interpretation and Technical Assistance. GL, JZ and CX conceived the study, analyzed the data, and wrote the manuscript. All authors read and approved the final manuscript.

## Conflict of Interest

The authors declare that the research was conducted in the absence of any commercial or financial relationships that could be construed as a potential conflict of interest.

## Publisher’s Note

All claims expressed in this article are solely those of the authors and do not necessarily represent those of their affiliated organizations, or those of the publisher, the editors and the reviewers. Any product that may be evaluated in this article, or claim that may be made by its manufacturer, is not guaranteed or endorsed by the publisher.
